# Assessment of Noise Levels and Perceptions of Its Health Impact at Kejetia Market in Ghana

**DOI:** 10.1155/2024/7658837

**Published:** 2024-06-26

**Authors:** Lyndon N. A. Sackey, Lawrencia S. Y. Agyemang, Patrick E. Acheampong, Michael Afriyie Owusu, Jennifer Amoah

**Affiliations:** Department of Environmental Science Kwame Nkrumah University of Science and Technology, Kumasi, Ghana

## Abstract

Noise pollution in developing countries such as Nigeria and Ghana is causing adverse effects on citizens, including hearing impairment, sleep disturbances, adverse social behavior, and cardiovascular diseases. This study assessed noise levels at the Kejetia Market in Ghana and the perceptions of health impact. A sound level meter (JD-801A) was used to measure the noise levels at the various points in the market. Results showed that noise exposure levels were not within Ghana Environmental Protection Agency standards 2008, with sources including loud music, advertisements, human congestion, and vehicles. Respondents perceived noise pollution sources as annoyance, mental stress, sleep disturbances, lack of concentration, hearing, and cardiovascular effects. The study suggests that stakeholders and authorities should educate the public on the health effects of noise pollution.

## 1. Introduction

Noise pollution is a significant environmental issue in urban and rural communities in developing countries such as Nigeria and Ghana [1]. Industrialization and urbanization have increased human activities, resulting in noise pollution. Periodic markets, where buyers and vendors gather for commercial transactions, create noise pollution [2]. Large crowds and loudspeakers contribute to noise pollution. Herbal medicine vendors also contribute to noise pollution. Small power generators and traffic create noise hazards. Occupational Safety and Health Act (OSHA) warns that prolonged noise exposure can lead to physical, physiological, and psychological issues such as hearing loss, speech interference, productivity reduction, increased blood pressure, and concentration loss [3].

Noise pollution is a significant issue in urban areas worldwide, with the World Health Organization (WHO) identifying it as the third most hazardous pollutant after air and water pollution [4]. The European Union estimates that over 40% of the population is exposed to a Day-Night of 55 dB or higher noise level, while 30% of the population is exposed to the same noise level during nighttime [5]. Recent studies show that 5 dB roadside noise increases hypertension risk by 3.4%, leading to hormonal dysfunction and blood pressure issues, affecting the cardiovascular system [6, 7].

The WHO identified seven categories of noise-related health impacts in 2011, including sleep disturbance, cardiovascular disease, learning impairment, and speech interference. Natural and manmade sources contribute to higher-level noise exposure, including traffic, industry, construction work, volcano eruptions, and thunder [8]. The Noise Observation and Information Service for Europe (NOISE) reports that road vehicle traffic generates the most noise exposure, with urban road traffic near infrastructures such as schools, offices, and residential buildings having the highest exposure levels [5, 9].

The Ghana Environmental Protection Agency (EPA) has launched a public education campaign to raise awareness about the harmful effects of noise pollution. The agency has set ambient noise level guidelines for residential areas with negligible transportation, stating that levels above 45 dB can impair sleep, those above 70 dB can cause emotional upset, and those above 90 dB can cause ear damage. Noise can also affect the circulatory, digestive, and nervous systems and vision. There are three broad categories of noise: transport noise, occupational noise, and neighborhood noise [10].

Civilization has increased sound levels, causing noise pollution to become a major concern for both the public and policymakers [11]. The EPA has set ambient noise levels to control its impact on human health, but the health effects of hazardous noise exposure are now considered an increasingly important public health problem. In Ghana, noise pollution is on the rise, with cities like Kumasi being the worst affected. Research has been conducted on noise pollution from religious establishments, as the building of religious meeting places in residential neighborhoods is widespread. Markets in big cities are overshadowed by various noise-generating activities, polluting the environment and affecting people at the market.

Noise is a significant environmental pollutant, causing health hazards and communication issues in the human environment. It is increasingly recognized as a public health issue, with marketplaces being no exception. Market noise is a major environmental pollutant that directly affects human performance and quality of exposure [12]. Persistent exposure to high noise levels can have serious health effects on customers and traders. This research aims to determine the noise levels at the market and ascertain the people's perceptions of noise generated at the Kejetia Market in Ghana. This will assist decision makers and policymakers in putting in measures to reduce or prevent noise pollution. Also, it could help in creating awareness of the health impact of noise pollution on human beings.

## 2. Materials and Methods

### 2.1. Study Area

**Figure 1:**
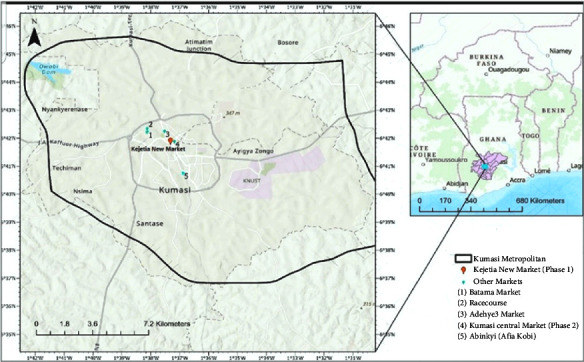
Map of Kumasi central market.

The study focused on Kumasi Central Market, also known as Kejetia Market, the largest single market in West Africa [13]. The market has three floors, with most facilities on the ground floor, including banks, clinics, transport systems, butcher's yards, police stations, and administration. The first and second floors have similar features, including restaurants, stores, and tailor's yards. The market covers 172,197 m^2^ with over 10,000 stores and stalls, with an average temperature of 24°C and humidity of 85% [13] (Figure 1).

### 2.2. Research Design

This study used a mixed research method, collecting quantitative data through noise level measurement using a sound level meter (JD-801A) and conducting qualitative interviews with market stakeholders.

### 2.3. Methods of Data Collection

#### 2.3.1. Quantitative Data Collection methods Survey Design

Sampling stations were chosen based on noise pollution levels on the ground, first, and second floors, as depicted in Table 1. The study focused on various areas in the building; the ground floor included the common areas, banks, butcher's yards, tailor yards, parking lots, mosque area, transport areas, and police stations. The first floor also included a restaurant, utility area, and common areas, while the second floor included walkways, tailor's yards, common areas, and a restaurant. Noise levels were measured for two weeks.

The noise level readings were taken using sound level meters (JD-801A). Calibration was done before sampling. Noise levels were measured at 30 locations, and the measurements were also recorded at 60-second intervals for 3 min. at 1.2 meters above the ground level. Temperature and humidity data were measured.

#### 2.3.2. Qualitative Data Collection Method

The study used open-ended questionnaires to gather qualitative data on noise pollution in the markets. Respondents were chosen from shops and offices in the markets and other stakeholders. Observation methods were employed to assess the situation, focusing on market settings, congested roads, religious activities, shops, and buildings using an observation checklist.

The questionnaire was structured to assess the general knowledge of noise, perception of noise sources, awareness of noise's impact on health, and mitigation action. The sample size for the research was 100, and this was determined by using the Slovin formula as shown in the following equation:

(1) $$\begin{array}{c} \text{Slovin formula}\left(n\right)=\frac{N}{\left(1+{Ne}^{2}\right)}, \end{array}$$

where *n* = number of samples, *N* = total population and *e* = error level confidence level of 95%, alpha level of 0.05.

### 2.4. Sampling Technique and Data Processing

The study utilized a sound level device (JD-801A) to measure the various sound levels at different locations of the market and the noise level compared with Ghana EPA guidelines, computing average noise levels for each location. Questionnaires were checked and sorted before data were entered into Special Package for Social Sciences (SPSS) software (version 27), considering humidity and temperature. Results represent exposure values at the same site. The sound levels were measured in triplicates.

### 2.5. Statistical Analysis

The study used SPSS version 27 for analyzing interview responses and Microsoft Excel 2010 for gathering and analyzing recorded noise levels. Analysis of variance (ANOVA) was used to calculate variance in the market noise levels.

## 3. Results

The Environmental Protection Agency (EPA) in Ghana has established guidelines for ambient noise levels, categorized into seven zones with specific day and night noise reception conditions, as shown in Table 2.

### 3.1. Sampling Sites

The study analyzed the ambient sound levels at various points in the market as shown in Table 1, to provide a comprehensive view of the sound levels within the market enclave.

### 3.2. Noise Levels at the Market

Tables 3, 4, 5, and 6 display average noise levels for different floors over two weeks, including weekly and overall averages.

Table 6 shows the descriptive analysis of the mean and standard deviation for the overall average noise levels for the two weeks for morning, afternoon, and evening.

### 3.3. Assessment of Noise Levels

**Figure 2:**
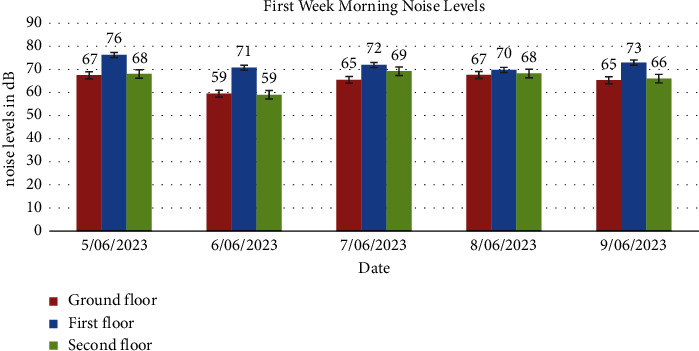
Average noise levels in the morning (6:00am-7:00am) of the first week for the floors.

**Figure 3:**
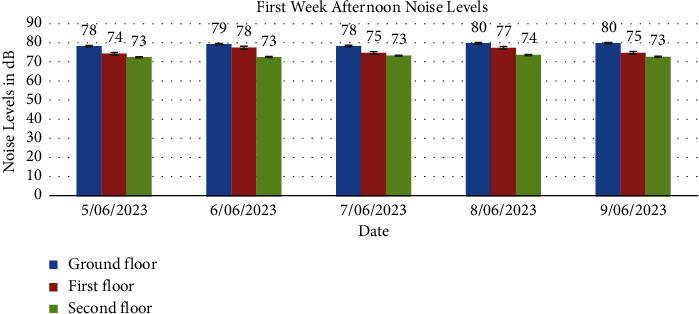
The average noise levels in the afternoon (12:00pm-1:00pm) of the first week for the ground floor, first floor, and second floor.

**Figure 4:**
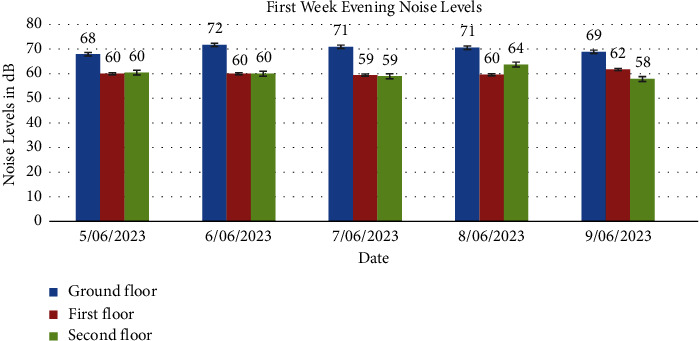
The average noise levels in the evening (6:00pm-7:00pm) of the first week for the ground floor, first floor, and second floor.

**Figure 5:**
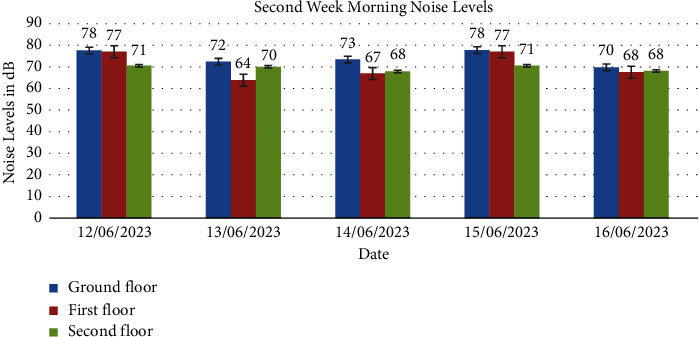
The average noise levels in the morning (6:00am-7:00am) of the second week for the ground floor, first floor, and second floor.

**Figure 6:**
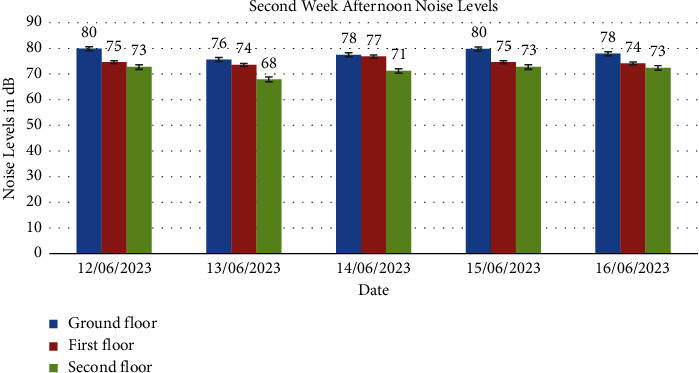
Average noise levels in the afternoon (12:00pm-1:00pm) of the second week for the floors.

**Figure 7:**
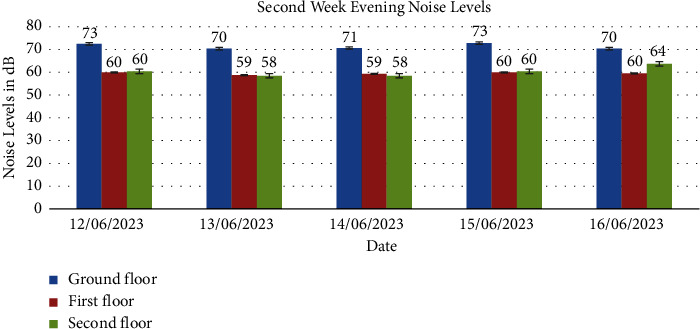
The average noise levels in the evening (6:00pm-7:00pm) of the second week for the ground floor, first floor, and second floor.

Noise levels obtained from the ground floor, first floor, and second floor for the two weeks are presented in Figures 1, 2, 3, 4, 5, 6, and 7.

### 3.4. Source of Noise Pollution

A survey study was conducted to gather feedback on major sources of market noise pollution, with the results presented in Table 7.

### 3.5. Assessment of the Perception of the Market People on Noise Pollution Impact on People in the Market

A survey study was conducted to assess the effects of noise pollution on market residents, with 100 respondents completing the questionnaires in Table 8.

## 4. Discussion

Noise exposure in 23 locations in the Kejetia Market showed variations in noise levels at different sampling positions, increasing or decreasing based on location. This indicates that the noise level cannot be the same at different locations because of factors such as weather, temperature, density of people, the wind direction, and the period of the day. The average noise levels during the first and second weeks were 68–70 dBA in the morning, 71–77 dBA in the afternoon, and 58–69 dBA in the evening. This implies that high levels of noise are produced in the market during the afternoon because usually that is when we have a lot of customers, movement of vehicles, and other peculiar activities of a market which generate a lot of noise. The average noise levels varied based on the sampling position. The study found that the second floor had the lowest noise levels during the day, while the ground floor recorded the highest noise levels during the day and night. The ground floor was generally higher than the other floors due to its significant market activities, mostly from mid-morning to afternoon. The ground floor is always congested with people, leading to increased temperature and humidity, potentially causing higher average noise levels [14]. However, daytime noise exposure is longer than nighttime exposure, with the average duration being higher. The sellers and customers who frequent the market stand a chance of becoming affected by different health issues. Long-term noise exposure is associated with cardiovascular disease (CVD), including acute cardiovascular events such as myocardial infarction and stroke [15].

The second floor's average noise exposure levels for day and night were below the EPA 2008 guidelines in Ghana, indicating less congested areas and fewer speakers used for product advertising. The first floor recorded higher noise levels than the permissible limit set by EPA (2008) during the day but within the limit during the night, possibly due to its proximity to the ground floor. However, the ground floor recorded higher noise levels than the EPA 2008 maximum permissible limit for day and night. The ground floor is primarily used for market activities due to high traffic and loud music used for product advertising. This study also found high variability in noise levels at different times of the day, with a standard deviation of 2.000, 3.000, and 6.350, respectively. The *p* value was less than 0.05 indicating a significant difference in average noise exposure levels during the day and night. Therefore, the noise generated at each floor influences the other floors. Hence, finding a way to reduce the noise pollution at the market must be done holistically and not just focusing on a floor or location in the market.

The study found that the main sources of noise in the market are loudspeakers used for product advertisement, human congestion, and vehicles, with the most prominent source being these speakers (82%), primarily used by vendors on the ground floor for advertisement (Table 6). Assessment of noise pollution in selected locations in Ota, Nigeria, reported similar sources of noise pollution originating from the market [16].

The study found that 82% of respondents considered noise a nuisance, with many annoyed by market activities while 5% of the people were indifferent. The noise levels were higher than the recommended standards and had been found to have unhealthy effects, as acknowledged by the respondents during the interview [1]. The ground floor produced the highest level of noise during the day (77 dB), causing high levels of annoyance. 80% agreed that noise can cause mental stress, 62% stated that it can affect sleep, 83% stated that it can affect concentration at work, and 37% stated that it can cause cardiovascular diseases. Gwanshak et al. [17] reported that noise has been identified to be a silent killer, yet not much has been done to control its impact, particularly in commercial centres of developing countries. The study highlights the importance of addressing noise pollution in urban areas. It was observed from the data collected that hearing effects were over 90%, primarily due to persistent noise from loudspeakers in the market, and a lack of concentration (83%), due to long-term exposure to noise [17]. These health impacts are attributed to the long duration of noise exposure at the market (Table 7). According to Chen et al. [18], a review reported that a long duration of noise exposure can cause adverse cardiovascular disease and mortality, diabetes, hearing impairment, neurological disorders, and adverse reproductive outcomes with environmental noise exposure in humans, especially occupational noise. Passchier-Vermeer and Passchier [19] reported that exposure to noise constitutes a health risk. There is sufficient scientific evidence that noise exposure can induce hearing impairment, hypertension and ischemic heart disease, annoyance, and sleep disturbance. This indicates that customers and vendors must be protected from excess noise pollution during their stay at the market to prevent any health impact.

The first and second floors had the lowest nightly noise level (58 dB), but most residents found it annoying due to its prolonged duration. Berglund et al. [20] and Lercher et al. [21] also reported that noise's ability to annoy depends on its physical characteristics, including sound pressure and spectral characteristics, and variations over time. Noise reactions are sensitive to social, psychological, and economic factors, and individual reactions vary significantly [22, 23]. Peak noise levels are recorded during the day, while lower levels occur at night. 78% of respondents believe loudspeaker noise should be stopped or minimized, while 20% believe it is unnecessary. A further 2% were noncommittal. Generally, the sources of noise pollution at the market must be managed to prevent any health impact on patrons of the market and the vendors. A noise pollution campaign can be embarked upon by the municipalities and other responsible agencies and authorities to educate the people on the health impact of noise pollution.

## 5. Conclusion and Future Perspective

In conclusion, the noise levels were generally high during the day and low at night at the market. The highest noise exposure levels were recorded in the afternoon (71 dB–77 dB), followed by noise levels in the morning (60 dB–70 dB) and the lowest noise levels in the evening (58 dB–66 dB). Also, the average noise exposure levels were found to be above the EPA permissible limit for the ground floor (day and night) and the first floor for the day. The second floor recorded the lowest for the two weeks. Overall noise levels were within the EPA permissible limits for day and night. There was variability in the levels of noise at various times of the day, which were high (afternoon—74 dB, morning—65 dB, and evening—62 dB). Noise generated by loud music and loudspeakers used to advertise products, human congestion, and vehicles were the major sources of noise pollution at the market. Respondents' perceptions of noise impact were annoyance, mental stress, effect on sleep, lack of concentration, effect on hearing, and cardiovascular effects. Market management should institute control measures to manage noise levels in the market. Safety sensitization programs should be organized to educate the populace on the health effects of noise pollution. Task forces should be organized to enforce the laws and monitor noise levels at the market.

## Figures and Tables

**Table 1:** Respective sampling areas at the Kejetia Market.

Sn	Sampling sites	Area	Category area
S1	Entrance (Gate 8)	Ground floor	Commercial
S2	Common area (Local foods, local Forex, Anwiam Clinic)		
S3	Bank (Fidelity Bank, Yaa Asantewaa and UBA)		
S4	Butcher's yard		
S5	Tailor yard 1 and 2		
S6	Parking lot		
S7	Mosque		
S8	Transport area (AB and CD)		
S9	Creche school		
S10	Rump area		
S11	Police station		
S12			

S13	Restaurant area	First floor	Commercial
S14	Common area (beads and tables)		
S15	Utility area		
S16	Common area (F0509–0532)		
S17	Common area (Clothing north)		
S18	Common area (Provision south)		

S19	Walkway (east and west)	Second floor	Commercial
S20	Tailor's yard		
S21	Common area (Clothing, north and south)		
S22	Common area (Tables s0211-0224)		
S23	Restaurant area		

**Table 2:** Ambient noise level guidelines for Environmental Protection Agency, EPA, Ghana.

Zone	Description of area of noise description	Permissible noise level in dB (A)	
		Day 06600-2200	Night 2200-0600
A	Residential areas with negligible or infrequent transportation	55	48
B1	Educational (school) and health (hospital) facilities	55	50
B2	Area with some commercial or light industry	60	55
C1	Area with some light industry, places of entertainment or public assembly, and places of worship such as churches and mosques	65	60
C2	Predominantly commercial areas	75	65
D	Light industrial areas	70	60
E	Predominantly heavy industrial areas	70	70

Source: Ghana EPA 2008.

**Table 3:** Average noise exposure levels for the first week.

Area	Morning (6:00am-7:00am) (dB)	Afternoon (12:00pm-1:00pm) (dB)	Evening (6:00pm-7:00pm) (dB)
Ground floor	64.6	79.0	70.2
First floor	72.4	75.8	60.2
Second floor	66.0	73.2	60.2

**Table 4:** Average noise exposure for the second week.

Area	Morning (6:00am-7:00am) (dB)	Afternoon (12:00pm-1:00pm) (dB)	Evening (6:00pm-7:00pm) (dB)
Ground floor	72	76	69
First floor	69	73	58
Second floor	68	69	58

**Table 5:** Overall average noise levels for the two weeks.

Area	Morning (6:00am-7:00am) (dB)	Afternoon (12:00pm-1:00pm) (dB)	Evening (6:00pm-7:00pm) (dB)
Ground floor	68	77	69
First floor	70	74	58
Second floor	66	71	58

**Table 6:** Descriptive analysis of the mean and standard deviation (SD) for the overall average noise levels for the two weeks.

Period of the day	Mean	SD
Morning (6:00am-7:00am)	68	2.000
Afternoon (12:00pm-1:00pm)	72	3.000
Evening (6:00pm-7:00pm)	62	6.350

Confidence level = 95% (*p* value < 0.05).

**Table 7:** Perception of people on the source of noise pollution at the market (*n* (%)).

Group	*N*	Loud music	Human congestion	Loudspeakers	Vehicle noise
Group 1	50	40 (80)	38 (76)	28 (56)	36 (78)
Group 2	40	35 (88)	15 (38)	30 (75)	20 (50)
Group 3	10	7 (60)	5 (50)	8 (80)	7 (70)
Total	100	82 (82)	58 (58)	66 (66)	63 (63)

Group 1 represents traders and hawkers randomly selected on the ground floor; Group 2 represents people on the first and second floors; Group 3 represents people at the management office of the market.

**Table 8:** Perception of people on the impact of noise pollution on health (*n* (%)).

Group	*n*	Annoyance	Effect on mental stress	Effect on sleep	Effect on hearing	Lack of concentration	Cardiovascular effects
Group 1	50	43 (86)	40 (80)	35 (70)	46 (92)	45 (90)	20 (40)
Group 2	40	34 (72)	32 (80)	20 (50)	35 (88)	30 (75)	10 (25)
Group 3	10	5 (50)	8 (80)	7 (70)	9 (90)	8 (80)	7 (70)
Total	100	82 (82)	80 (80)	62 (62)	90 (90)	83 (83)	37 (37)

Group 1 represents traders and hackers randomly selected on the ground floor; Group 2 represents people on the first and second floors; Group 3 represents people at the management office of the market.

## Data Availability

Data are available upon request.
